# An expanded age range for meningococcal meningitis: molecular diagnostic evidence from population-based surveillance in Asia

**DOI:** 10.1186/1471-2334-12-310

**Published:** 2012-11-19

**Authors:** Soon Ae Kim, Dong Wook Kim, Bai Qing Dong, Jung Soo Kim, Dang Duc Anh, Paul E Kilgore

**Affiliations:** 1 Translational Research Division, International Vaccine Institute, SNU Research Park, San 4-8 Nakseongdae-Dong, Kwanak Gu, Seoul, 151-919, South Korea; 2 Laboratory Science Division, International Vaccine Institute, Seoul, South Korea; 3 Guangxi Zhuang Autonomous Region Center for Disease Control & Prevention, Nanning, China; 4 Department of Pediatrics, Jeonbuk National University Hospital, Jeonju, South Korea; 5 National Institute of Hygiene and Epidemiology, Hanoi, Vietnam; 6 Department of Pharmacy, College of Pharmacy, Hanyang University, Suwon, South Korea; 7 Department of Pharmacy Practice, Eugene Applebaum College of Pharmacy & Health Sciences, Wayne State University, Detroit, USA

**Keywords:** Cerebrospinal fluid, Meningococcal meningitis, *Neisseria meningitidis*, Serogroup, Surveillance

## Abstract

**Background:**

To understand epidemiologic patterns of meningococcal disease in Asia, we performed a retrospective molecular analysis of cerebrospinal fluid (CSF) specimens collected in prospective surveillance among children aged < 5 years of age in China, South Korea, and Vietnam.

**Methods:**

A total of 295 isolates and 2,302 CSFs were tested by a meningococcal species- and serogroup-specific polymerase chain reaction (PCR) assay targeting the *Neisseria meningitidis* (Nm) *ctrA* gene. Multi-locus sequence typing (MLST) was performed in Nm gene amplification analysis and incidence rates for meningococcal meningitis were estimated.

**Results:**

Among 295 isolates tested, 10 specimens from Vietnam were confirmed as serogroup B and all were Sequence Type (ST) 1576 by MLST. Among the 2,032 CSF specimen tested, 284 (14%) were confirmed by PCR (*ctrA* gene), including 67 (23.6%) from China, 92 (32.4%) from Korea, and 125 (44.0%) from Vietnam. Neonates and infants aged < 6 months of age accounted for more than 50% of Nm-PCR positive CSF. Two CSF specimens from Vietnam were identified as serogroup B using MLST. In addition, 44 specimens underwent sequencing to confirm meningococcal serogroup; of these, 21 (48%) were serogroup C, 12 (27%) were serogroup X, 9 (20%) were serogroup Y and 2 (5%) were serogroup B. The incidence rates of meningococcal meningitis among children < 5 years of age was highest in Vietnam (7.4/100,000 [95% CI, 3.6—15.3] followed by Korea (6.8/100,000 [95% CI, 3.5-13.5] and China (2.1/100,000) [95% CI, 0.7-6.2]).

**Conclusions:**

These results suggest that there is a previously undetected, yet substantial burden of meningococcal meningitis among infants and young children. Standardized, sensitive and specific molecular diagnostic assays with Nm serogrouping capacity are needed throughout Asia to understand the true burden of *N. meningitidis* disease.

## Background

*Neisseria meningitidis* (*N. meningitidis*) is a gram-negative, encapsulated *β*-proteobacterium and the leading cause of epidemic meningitis. Globally, *N. meningitidis* causes an estimated 1.2 million cases and 135,000 deaths each year [1-3]. In Africa, *N. meningitidis* is the leading cause of severe, life-threatening meningitis and is responsible for thousands of cases and scores of deaths across sub-Saharan “meningitis belt” countries [4-7]. Moreover, *N. meningitidis* serogroups A and W-135 have been exported from Asia to other regions [8]. Previous studies suggest that these meningococcal serogroups were carried to the Middle East/Mediterranean region where meningococcal disease outbreaks subsequently occurred among Hajj pilgrims and across sub-Saharan African countries [4,9-12].

In Asia, meningitis outbreaks have been reported in child-care settings, schools, college dormitories, military camps, and among returning Hajj pilgrims [8,10,12-15]. Despite these reports documenting sporadic and epidemic meningococcal disease, there are large gaps in our knowledge of meningococcal disease patterns across large populations of Asia. It is believed that vaccine introduction against meningococcal disease may have been an important factor leading to the control of meningococcal epidemics [16,17], yet there is limited introduction of vaccines against meningococcal meningitis in Asian countries [13]. In addition, the serogroup distribution of meningococcus in Asia is scarce, in part, due to the limited availability of appropriate diagnostic tests including molecular assays [13,18]. Here, through the application of molecular analyses, we provide new evidence of meningococcal meningitis among hospitalized children < 5 years of age who were identified during prospective, population-based surveillance in China, South Korea, and Vietnam.

## Methods

### Study populations and surveillance

In three study sites, prospective, population-based surveillance for invasive bacterial meningitis in children < 5 years of age was conducted over a 2-year period. These sites were located in the Nanning, China (January 2001 - December 2002), the Jeonbuk Province, South Korea (September 1999 - December 2001), and Hanoi, Vietnam (March 2000 - February 2002). In China, children residing in Nanning, Wuming and Yongning counties of the Guangxi Province were referred to one of six study hospitals if they had clinical signs and symptoms of bacterial meningitis. In Jeonbuk province, located in the southwestern region of South Korea, study sites included the provincial capital city of Jeonju and 10 districts within the province. In Vietnam, children who resided in any of the seven urban districts of Hanoi were referred to one of three central hospitals in Hanoi for study enrollment. Following specimen collection, cerebrospinal fluid (CSF) specimens were streaked on commercial blood agar culture media (Becton, Dickinson & Co., Franklin Lakes, USA), incubated in 5% CO_2_ at 37°C for 3 days and checked daily for bacterial growth. After completion of routine biochemical, hematological and microbiological tests, CSF specimens were preserved at −70°C for further molecular analysis at the time surveillance conduction. Detailed methods for these standardized surveillance studies have been previously described [19-21]. For the analysis of specimens in this study, we utilized CSF specimens preserved from the previously conducted surveillance study [19-21]. All CSF specimens utilized in this study were de-identified prior to laboratory processing and analysis. In the context of the population-based surveillance study of meningitis conducted from 1999 to 2002, written consent was not obtained as the collection of CSF was considered routine standard of care for hospitalized children with suspected bacterial meningitis. For this reason, verbal consent of the parent or legal guardian present with the child during the hospitalization was recorded in the medical chart of the patient at the time of the clinical lumbar puncture procedure. This consenting procedure was approved by the local scientific ethical review committees of participating institutions. Ethical approvals for patient specimen collection during surveillance were obtained from the following institutions: International Vaccine Institute, Seoul, Korea; Harbor UCLA Medical Center, Torrance, CA, USA; Jeonbuk National University Hospital, Jeonju, Korea; Chonju Presbyterian Hospital, Jeonju, Korea; Namwon Medical Center, Namwon, Korea; Jeongeub Asan Foundation Hospital, Jeongeub, Korea; Won Kwan University Hospital, Iksan, Korea; National Institute of Hygiene and Epidemiology, Hanoi, Vietnam; National Institute of Pediatrics, Hanoi, Vietnam; St. Paul Hospital, Hanoi, Vietnam; Bach Mai Hospital, Hanoi, Vietnam and the Guangxi Zhuang Autonomous Region Center for Disease Control, Nanning, China.

### Laboratory testing

The preserved, frozen CSFs and bacterial isolates that were collected during the prospective meningitis surveillance studies were used for the present analyses. Prior to molecular analysis of CSF specimens, isolates were sub-cultured to confirm the bacterial species present in each specimen. *N. meningitidis* identification and serogroup identification were conducted for all bacterial isolates using multiplex polymerase chain reaction (PCR) targeting the *ctrA* gene [22]. PCR primers used for identification of three bacteria *(S. pneumoniae*, *Haemophilus influenzae* type b (Hib)*,* and *N. meningitidis*) were *ctrA* primer sequences from Corless, *et al*. [23]. HinBF and HinBR primers for detection of *H. influenzae* type b using specific region of *bexA* gene were designed based on the report by Corless, *et al*. [23]. *lytA* primer sequences were modified based on a previous report by Llull, *et al*. [24]. PCR detection sensitivity of *ctrA* gene was validated by PCR reaction of serially-diluted reference DNA. Genomic DNA was prepared from bacterial culture as described previously [25] and DNA concentration was determined with a Nano Drop spectrophotometer. The genomic DNA prep concentration was adjusted to 100 ng/μl and serially-diluted ten-fold to 1 pg/μl. Based on the genome size of three serogroups of *N. meningitidis* (serogroup A, strain z2491: 2,184,406 bp, serogroup B: 2,272,351 bp, and serogroup C, strain FAM18: 2,194,961 bp), the molecular weight of a single copy of *N. meningitidis* genome was calculated as 2 fg/one genome copy. Two microliters of each diluted reference DNA were used for template of the PCR reaction. *N. meningitidis* genomic DNA was purified from the bacterial cultures of *N. meningitidis*-confirmed isolates. The genomic DNA was further analyzed by two serogroup identification methods based on the *N. meningitidis* sialyltransferase (*sia*D) gene and serogroup-specific *syn* gene complex [22,26]. Primers used for serotyping of *N. meningitidis* A, B, C, Y, and W135 were designed at the International Vaccine Institute (Additional file 1: Table S1).

The CSF samples were processed as previously described [25]. Briefly, 200 μl of each CSF sample was boiled for 3 minutes and then centrifuged for 5 minutes at 9,000 × *g*. The supernatant was removed and put into a new tube, and 2 μl of the supernatant was used as the template in the 25 μl PCR reaction. *N. meningitidis* PCRs targeting the *cr*gA, IS*1106*, and 16S rRNA genes were performed on all MLST-negative CSF specimens to identify additional *N. meningitidis*-positive CSF specimens. The established multi-locus sequence typing (MLST) scheme ( http://pubmlst.org/neisseria/) for *Neisseria spp.* was used to analyze bacterial isolates and CSF specimens that were positive for *N. meningitidis* specific gene amplification analysis. *N. meningitidis*-specific gene amplification analysis of *N. meningitidis* PCR-positive CSF with the presence of contact-regulated gene A (*cr*gA), IS1106, and 16S rRNA was conducted.

### Data collection and statistical analysis

For analysis of epidemiologic data, the original study surveillance database from each field site was merged with *N. meningitidis* laboratory results using a unique study identification number available for each patient. For age group analysis, patients were grouped into the following age strata: < 1, 1–6, 7–11, 12–23, 24–35, 36–47, and 48–59 months of age. Children with PCR-confirmed *N. meningitidis* were grouped to describe demographic characteristics, clinical signs and symptoms of meningitis, prior use of antibiotics and outcomes at discharge. Further analysis of results from *N. meningitidis* serogroup-specific PCR and sequencing results were used to describe patient distributions by clinical characteristics and month of admission. To estimate the incidence of serogrouped *N. meningitidis*, previously published population denominator data for children < 5 years of age were applied for the calculations of incidence rates [19-21]. Ninety-five percent confidence limits for incidence rates were calculated using the Wilson score method [27]. Data analysis was performed using STATA (version 11.0, StataCorp LP, TX, USA).

## Results

### Demographic and clinical characteristics

A total of 2,327 specimens (295 isolates and 2,032 CSF) collected from children in China, 626 (26.9%), Korea, 700 (30.1%) and Vietnam, 1001 (43.0%) were processed for nucleic acid testing. Male patients constituted 63.6% of enrolled subjects. Among children aged < 5 years, more than half (n = 1,392) were infants aged < 1 year (including 332 who were < 1 month of age, 772 aged 1–6 months, and 288 aged 7–11 months). Among 295 bacterial isolates, 10 isolates in Vietnam were confirmed as *N. meningitidis*. Of these, one patient was younger than one month old, one was 1–6 months of age, four were 7–11 months of age, and four patients were 12–35 months of age. Five of the culture-confirmed patients were male and half were female. Among the 2,032 cerebrospinal fluid (CSF) specimens tested, 14% (n = 284) were confirmed by *N. meningitidis* PCR targeting the *ctrA* gene, including 67 patients (11.4%) in China, 92 (13.2%) in Korea, and 125 (14.2%) in Vietnam. *S. pneumoniae* and *Haemphilus influenzae* type b (Hib) were identified as well (Table 1). Among those with *N. meningitidis* PCR-confirmed infection, 26 specimens (11 in China and 15 in Vietnam) could not be linked with the original clinical database due to missing variables, resulting in a total of 258 meningococcal meningitis patients with complete data available for further analysis.

**Table 1:** Cerebrospinal fluid analysis of simultaneous detection of bacteria by PCR

**Bacteria**	**China**	**Korea**	**Vietnam**	**Total**
	**Number (%)**	**Number (%)**	**Number (%)**	
*S. pneumoniae*	27 (4.6)	29 (4.2)	41 (4.7)	97
*Haemophilus Influenzae* type b	12 (2.0)	38 (5.5)	146 (16.6)	196
*N. meningitidis*	67 (11.4)	92 (13.2)	125 (14.2)	284
None Detected	511 (87.0)	538 (77.1)	566 (64.5)	1615
Total	587 (100)*	608 (100)*	837 (100)*	2032

*30 (China), 89 (Korea), 41 (Vietnam) CSF specimens were positive for more than two bacterial species; **total number was subtracted 160 from 2192 that was more than two bacterial species among three countries.

Overall, the mean age of children with *N. meningitidis* PCR-confirmed meningitis was 14.4 (± 17.7) months, with a mean age of 17.4 (± 19.2) months among females *versus* 12.6 (± 16.5) months among in males (*P* < 0.05). For all countries combined with culture confirmed and PCR-confirmed, males accounted for 61.9% (n = 166) and females were 38.1% (n = 102, *P* < 0.05). Approximately half of the *N. meningitidis* PCR-confirmed patients were < 6 months of age (50.0% in China; 58.7% in Korea; and 47.3% in Vietnam), and more than three-quarters (75.6%) of them were young children aged < 2 years old (Table 2).

**Table 2:** Meningococcal meningitis among children aged < 60 months in China, Korea and Vietnam by age and gender group, 1999 through 2002*

**Characteristics**	**PCR-confirmed**			**Culture-confirmed**	**Total**
	**China**	**Korea**	**Vietnam**	**Vietnam**	
	**(n = 56)**	**(n = 92)**	**(n = 110)**	**(n = 10)**	**(n = 268)**^**b**^
	**N (%)**	**N (%)**	**N (%)**	**N (%)**	**N (%)**
Age (months)					
< 1	17 (30.4)	28 (30.4)	23 (20.9)	1 (10.0)	69 (25.7)
1-6	11 (19.6)	26 (28.3)	29 (26.4)	1 (10.0)	67 (25.0)
7-11	5 (8.9)	4 (4.3)	21 (19.1)	4 (40.0)	34 (12.7)
12-23	11 (19.6)	11 (12.0)	9 (8.2)	3 (30.0)	34 (12.7)
24-35	3 (5.4)	4 (4.3)	8 (7.3)	1 (10.0)	16 (6.0)
36-47	6 (10.7)	11 (12.0)	11 (10.0)	0	28 (10.4)
48-59	3 (5.4)	8 (8.7)	9 (8.2)	0	20 (7.5)
**Gender**					
Female	13 (23.2)	31 (33.7)	53 (48.2)	5 (50.0)	102 (38.1)
Male	43 (76.8)	61 (66.3)	57 (51.8)	5 (50.0)	166 (61.9)
**Clinical features**					
Lethargy/coma	19 (33.9)	8 (8.7)	60 (54.5)	4 (40.0)	91 (34.0)
Irritability/confusion	17 (30.4)	7 (7.6)	29 (26.4)	4 (40.0)	57 (21.3)
Nuchal rigidity	17 (30.4)	7 (7.6)	43 (39.1)	6 (60.0)	71 (26.5)
Seizure	30 (53.6)	13 (14.1)	51 (46.4)	4 (40.0)	98 (36.6)
Vomiting	3 (5.4)	18 (19.6)	64 (58.2)	7 (70.0)	92 (34.3)
Bulging fontanelle	8 (14.3)	1 (1.1)	31 (28.2)	2 (20.0)	42 (15.7)
Fever or septic appearance	29 (51.8)	80 (87.0)	82 (74.5)	9 (90.0)	200 (74.6)
**Outcome**					
Alive	53 (94.6)	UK	UK	8 (80.0)	NA
Deceased	3 (5.4)	UK	UK	2 (20.0)	NA
***Prior antibiotic usage***					
Yes	23 (41.1)	53 (57.6)	78 (70.9)	10 (100.0)	164 (61.2)

^a^Surveillance periods: China, January 1, 2000--December 31, 2002; South Korea: September 1, 1999--December 31, 2001; Vietnam: March 4, 2000-March 3, 2002.

^b^26 specimens (China, n = 11; Vietnam, n = 15) could not be linked with the clinical database were excluded from analysis.

Among patients who had PCR-confirmed meningitis, the mean length of their hospital stay was 8.2 days (± 11.3 SD; median stay: 6 days). At the time of initial clinical presentation, patients exhibited severe manifestations of meningitis including both fever and sepsis (74%; n = 191), followed by seizure (36.4%; n = 94), vomiting (33.0%; n = 85), lethargy or coma (34.0%; n = 87), stiff neck (26.0%; n = 67), irritability or confusion (20.5%; n = 53), and bulging fontanelle (16.0%; n = 40) (Table 2). In Korea and Vietnam, the majority (87% and 74.5%, respectively) of PCR-confirmed *N. meningitidis* patients were found to have fever and sepsis upon initial presentation, while this was less frequent (51.8%; n = 29) in China. Seizures were notably more common among Chinese children (53.6%; n = 30) compared with either Korean (14.1%; n = 13) or Vietnamese (46.4%; n = 51) children. Overall, 17.4% (n = 45) were hospitalized in the neonatal ward or intensive care unit (5.0%; n = 13), which might have been caused by severe clinical signs and symptoms. Among the *N. meningitidis* PCR-confirmed children, five deaths were observed. Three deaths were identified in China within the early days of life: one was identified at three days of age, another one at one week of age, and one at 3.5 weeks of age. Two other deaths were identified in Vietnam, where death occurred at two days of age and at 22.3 months of age.

During the original prospective surveillance studies, over-the-counter use of antibiotics without a prescription was common in each country. In Vietnam, 70.9% of *N. meningitidis* PCR-confirmed patients reported antibiotic consumption prior to hospital admission, compared with 57.6% in Korea and 41.4% in China (overall consumption of antibiotics among the children with meningococcal disease in the three sites including culture-confirmed patients, 61.2% [164/258]).

### Incidence rates

The incidence rates of *N. meningitidis* by culture and serogroup-specific PCR and sequencing was highest in Vietnam at 7.4/100,000 population (95% CI, 3.6-15.3), followed by Korea 6.8/100,000 (95% CI, 3.5-13.5) and China 2.1/100,000 (95% CI, 0.7-6.2) (Table 3). In China and Korea, age-specific incidence rates were highest in children aged < 6 months, whilst in Vietnam, rates were highest in those aged < 12 months. Of these, there was a particularly high incidence rate of meningococcal meningitis among very young infants aged less than one month of age: in China, Korea, and Vietnam incidence was 96/100,000 (95% CI, 29.7-309.4), 81.5/100,000 (95% CI, 18.8-352.2), and 36.2/100,000 (95% CI, 3.8-346.0), respectively. Consequently, the incidence rate was highest in children during the first year of life in all three countries (Table 3).

**Table 3:** **Incidence of meningococcal meningitis by culture (n = 10) and serogroup-specific PCR and sequencing (n = 43) among children aged < 5 years old in China, South Korea, and Vietnam by age group, 1999 through 2002**^**a**^

**Age (months)**		**China**		**Korea**		**Vietnam**
	**N**	**Incidence rate (95% CI)**	**N**	**Incidence rate (95% CI)**	**N**	**Incidence rate (95% CI)**
< 6	5	14.6 (4.5-47.2)	7	27.2 (9.9-74.2)	11	15.5 (3.6-67.2)
7-11	1	3.4 (0.4-33.0)	0	0	9	29.1 (8.0-106.0)
12-23	0	0	2	4.5 (0.8-25.8)	5	13.7 (3.8-50.1)
24-59	0	0	7	4.8 (1.8-13.1)	6	2.4 (0.5-10.2)
overall	6	2.1 (0.7-6.2)	16	6.8 (3.5-13.5)	31	7.4 (3.6-15.3)

^a^Surveillance periods: China, January 1, 2000--December 31, 2002; South Korea: September 1, 1999--December 31, 2001; Vietnam: March 4, 2000-March 3, 2002

^b^Incidence rates are shown per 100,000 children; 95% confidence intervals are shown in parentheses.

With respect to monthly distributions, we found that *N. meningitidis* cases increased during May through August in China and peaked sharply from March to June in Vietnam. In Korea, the number of *N. meningitidis* cases was highest from February through April (data not shown).

### PCR results and serogroup

All 10 meningococcal serogroup B among isolates originated from Vietnam and two CSF specimens from Vietnam contained the allele type 140, 5, 9, 173, 175, 34, 165 (in the order *abcZ*, *adk*, *aroE*, *fumC*, *gdh*, *pdhC*, and *pgm*), which has been designated as sequence type (ST) 1576 (Additional file 1: Tables S2 and S3). CSF specimens that were *N. meningitidis* PCR-positive for *ctrA* and positive for serogroup identification by PCR, but negative by MLST were further analyzed by PCR for the presence of *crgA*, IS*1106*, and *N. meningitidis* specific 16S rRNA.

**Figure 1:**
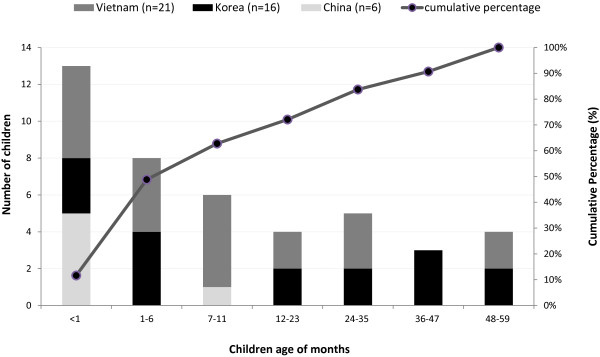
**Number and cumulative percentage of meningococcal meningitis from PCR confirmation with *ctrA *gene by age group among children aged < 5 years old in China, South Korea, and Vietnam by age group, 1999 through 2002**^**a**^**.**^a^ Surveillance periods: January 1, 2000-December 31, 2002, China; September 1, 1999-December 31, 2001 (4-month hiatus, August to November 2000), Korea; March 4, 2000-March 3, 2002, Vietnam.

A total of 44 *N. meningitidis* PCR-positive CSF were serogrouped, but one did not link to clinical data, resulting in 43 specimens with complete serogroup and clinical information available for further analysis. Of these, 20 (91%) CSFs from Vietnam were found to have *N. meningitidis* serogroup C, and two (9%) were positive for serogroup B. In contrast, serogroups X and Y were prevalent in China (50% [n = 3] X and 33% Y [n = 2]), while serogroup X comprised 56% [n = 9] and serogroup Y [n = 6] comprised 38% in Korea (Table 4). Eighty-four percent of the CSF specimens that were positive by the *N. meningitidis* PCR targeting the *ctrA* gene could not be serogrouped. Among the 43 children with *N. meningitidis* PCR-positive CSF and complete demographic data, 48.8% (n = 21) were less than six months of age (Figure 1). In China, among the 6 children whose specimens could be serogrouped, five were less than one month of age and one was < 12 months of age. Among the 16 *N. meningitidis* PCR-positive Korean children whose specimens could be serogrouped, seven were < 6 month of age and two were < 2 years of age, and the remaining seven were older than two years of age. In Vietnam, of the 21 *N. meningitidis* PCR-positive and serogrouped children, five were < 1 month of age, four were 1–6 months of age, five were <12 months of age, and seven children were older than one year of age (Figure 1).

**Table 4:** **Serogroup distributions of *N. Meningitidis *among Cerebrospinal fluid specimens and bacterial isolates among children aged < 5 years old in China, South Korea, and Vietnam by age group, 1999 through 2002**^**a**^

**Serogroup**	**Cerebrospinal fluid**			**Bacterial isolates**
	**China**	**Korea**	**Vietnam**	**Vietnam**
		**N (%)**		**N (%)**
A	0	0	0	0
B	0	0	2 (9.1)	10 (100)
C	0	1 (6.3)	20 (90.9)	0
W-135	1 (16.7)	0	0	0
X	3 (50.0)	9 (56.3)	0	0
Y	2 (33.3)	6 (37.5)	0	0
Total	6 (100)	16 (100)	22 (100) ^b^	10 (100)

^a^Surveillance periods: China, January 1, 2000--December 31, 2002; South Korea: September 1, 1999--December 31, 2001; Vietnam: March 4, 2000-March 3, 2002.

^b^One specimen could not be linked with the clinical database.

Further analyses were conducted. Two serogroup B positive CSF specimens in Vietnam also showed positive results in *N. meningitidis* MLST analysis. However, the 43 *N. meningitidis ctrA* positive CSF specimens that were also positive by serogrouping PCR assay showed negative results in the *N. meningitidis* MLST. Among the 20 *N. meningitidis* serogroup C positive CSF specimens collected in Vietnam, 16 were positive for *crgA* detection, 5 were positive for IS*1106* detection, and 2 showed positive results in detection of 16S rRNA (Additional file 1: Table S2).

## Discussion

This is the first and largest collection of CSF specimens obtained during population-based studies in representative populations of Asian children that provides laboratory-confirmed meningococcal meningitis incidence rates based on molecular diagnostic technologies. The application of modern molecular diagnostics for *N. meningitidis* identified case clusters of meningococcal serogroup B meningitis and an additional high number of serogroup X and Y meningococcal meningitis that had been previously unrecognized when CSFs were tested only by culture and antigen detection methods alone during the original surveillance study periods. Our findings suggest that retrospective molecular analyses of well-preserved clinical specimens in a biorepository provide novel insights into causes of childhood meningitis that are potentially vaccine-preventable diseases, particularly among patients who may commonly use antibiotics.

Based on the combined testing of CSFs by bacterial culture and PCR, this study demonstrated that 75.6% of the children identified with *N. meningitidis* in CSF were less than 2 years of age: this occurred particularly among infants less than 6 months of age and even in neonates. Previous incidence studies of *N. meningitidis* among children have demonstrated a sizable disease burden. For example, among children under 4 years of age in the United Kingdom [28], rates of serogroup B meningococcal infections peaked among infants aged less than one year of age with, accounting for almost 90% of laboratory confirmed cases. There is still no licensed vaccine available to protect against serogroup B disease and Active Bacterial Core surveillance (ABCs) data in the United States estimated that infants aged < 1 year had the highest rates of meningococcal disease at 5.38 cases per 100,000 population in 1998–2007 [29]. In Romania, children aged < 5 years old were found to have a meningococcal meningitis incidence rate of 22 per 100,000 annually based on prospective population-based surveillance in 2000–2002 [30].

In spite of reports of sporadic or outbreak-associated meningococcal disease in Asia, there are limited data on meningococcal incidence rates and on *N. meningitidis* serogroup distribution. In Korea, there is an estimated annual incidence of 2.2 per 100,000 among 550,000 soldiers [14]; a nationwide study among 17 university hospitals also reported bacteria-proven meningococcal meningitis in 125 cases from 1996 to 2005. Of these, two (1.6%) were less than one month of age [31]. Available study identified outbreaks of *N. meningitidis* in southern province in Vietnam [32].

Our findings describing the meningococcal serogroup distribution are inconsistent with previous studies. For example, there have been no reports on serogroup X in Asia in the past 50 years [13]. There is also a recent increase in serogroup B, C, and Y predominating in Europe, the United States, and Australia, whilst serogroup A is prevalent in Africa and Asia [12,13,33]. All of the bacterial isolates in Vietnam from our analysis were serotype B, which is the most commonly identified serogroup in infants in endemic settings. Prolonged outbreaks of serogroup B disease in some countries have also caused substantial morbidity and mortality [34,35] and there is presently no available vaccine against this serogroup [2,30,36]. The occurrence of a cluster of serogroup B meningococcal meningitis in Vietnam underscores the need for further investigation of epidemiologic factors and transmission dynamics that drive circulation of meningococcal serogroups. In addition, a substantial number of serogroup X and Y in China and Korea was identified in the present study, suggesting that serogroup X may have been previously under-detected due to limited surveillance or limited availability of assays capable of detecting serogroup X. Among the three countries, *N. meningitidis* was responsible for very large epidemics in China, leading to the development and introduction of serogroup A and C meningococcal polysaccharide vaccines for children starting in the 1980s [37]. Nonetheless and in spite of the vaccine having been introduced in China, there have been sporadic outbreaks and increased cases of circulating serogroup A and C infections in 2003–2006 [38]. There have also been continuous reports of serogroup B infections, which have been confirmed with modern molecular diagnostics [37-39]. In Korea, our results showed a unique serogroup distribution (serogroup X [9/16], Y [6/16] and C [1/16]), relative to a previous study that reported serogroup Y as the most frequently detected serogroup (9/11 isolates). Another study found one serogroup A and two serogroup C strains among ten *crg* A PCR positive specimens [14,40]. Current available vaccines target serogroups A, B, C, and W-135 [12,41], but do not cover serogroups X and Y. The results from our analysis suggest that existing knowledge gaps may be filled through the application of systematic surveillance that applies molecular diagnostic techniques for detection of *N. meningitidis*. The application of molecular testing for meningococcus has had a substantial impact in our understanding epidemiologic patterns of meningococcus in Asia and the potential role of contemporary available meningococcal vaccines.

Clinically, our CSF analysis confirms that infants and young age groups were particularly vulnerable to meningococcal meningitis, suffering severe signs and symptoms, including sepsis, seizure, coma and lethargy, and vomiting. Our findings are intriguing given the limited number of previous studies that have reported *N. meningitidis* infection in neonates and young infants [33,42,43]. These new results suggest the need to improve surveillance for meningitis among infants and to consider the role of meningococcal vaccines among infants [12,41].

Although this study provides one of the largest CSF analyses to describe *N. meningitidis* in Asian countries, there are some limitations. For example, 84.1% of the *N. meningitidis* positive CSF specimens could not be serogrouped and may have resulted from partial degradation of pathogen components during storage. In addition, some specimen testing may have been limited by prolonged storage that was associated with diminution of specimen volume through evaporation or prolonged storage. Second, although original studies were done in representative populations, rates of meningococcal meningitis among children in other parts of each country and in other countries in Asia may differ from those found in this analysis. Third, overall reduced detection of bacterial pathogens, including common invasive species causing meningitis in children, may, at least in part, be the result of widespread antibiotic overuse that was observed throughout each study site in China, Korea and Vietnam. In spite of the easy access to antibiotics in these three countries, an 11.6% fatality prevailed and remains poorly understood.

## Conclusions

The burden of *N. meningitidis*-associated meningitis among Asian children aged < 5 years old was found to be substantially higher than previously reported. Incidence rates among neonates and infants suggest that meningococcal meningitis may be affecting a younger age range of patients than previously understood. By understanding the dynamics of *N. meningitidis,* with incorporation of routine PCR methods for detection of *N. meningitidis* and the use of standardized surveillance methods for meningococcal meningitis, many countries will have the capacity to more accurately describe and mitigate invasive meningococcal disease. This may be of patients particularly true in areas with high antimicrobial usage in developing or middle income countries, where laboratory culture capacity may be limited. To date, more than a decade after the original prospective surveillance studies were completed, there have been few improvements in surveillance for *N. meningitidis* across Asia. In addition, given these observations, it is likely that new surveillance may uncover additional serogroups, including those identified in the results presented here. Therefore, we strongly advocate for a pan-Asian surveillance strategy focused on meningococcal meningitis to better understand the epidemiology and to implement prevention strategies (e.g., vaccines) in this region.

## Competing interests

The authors declare that they have no competing interests.

## Authors’ contributions

SAK and PEK carried out study design, data collection and analysis, and drafted the manuscript. DWK performed study design and conducted molecular laboratory test and drafted the manuscript. BQD, JSK and DDA participated in its design and helped to draft the manuscript. All authors read and approved the final manuscript.

## Pre-publication history

The pre-publication history for this paper can be accessed here:

http://www.biomedcentral.com/1471-2334/12/310/prepub

## Supplementary Material

Additional file 1 **Table S1.** Primers used for serotyping of *N. meningitidis* A, B, C, Y, and W135 (4)*. **S2**. *Neisseria meningitidis* identifications in CSF specimens, in China, South Korea, and Vietnam, 1999 through 2002. **S3**. Multi-locus sequence typing (MLST) results of 10 *N. meningitidis* isolates and 2 CSF specimens collected in Vietnam. Click here for file
