# Supplementary material for: An expanded age range for meningococcal meningitis: molecular diagnostic evidence from population-based surveillance in Asia
Source: BMC Infect Dis. 2012 Nov 19;12:310. doi: 10.1186/1471-2334-12-310 (PMC3519641; doi:10.1186/1471-2334-12-310)
Supplement: Additional file 1 — Table S1. Primers used for serotyping of N. meningitidis A, B, C, Y, and W135 (4)*. S2. Neisseria meningitidis identifications in CSF specimens, in China, South Korea, and Vietnam, 1999 through 2002. S3. Multi-locus sequence typing (MLST) results of 10 N. meningitidis isolates and 2 CSF specimens collected in Vietnam. [file 1471-2334-12-310-S1.doc]

**Additional Tables**

Additional. Table 1. Primers used for serotyping of *N. meningitidis* A, B, C, Y, and W135 (4)*.

| **Target** | **Sequences** | **Target size (bp)** |
| --- | --- | --- |
| mynB/sacB A F | AAG ATG ATG CTA GAG GCA CT | 243 |
| mynB/sacB A R | GGA TGG TTT TCG AGC GTG T |  |
|  |  |  |
| siaD(B)F | ACA CCA TTA CTC TCA CCC TCA AC | 103 |
| siaD(B)R | CTT GGA TCA TTT CAG TGT TTT CCA C |  |
|  |  |  |
| siaD(C)F | TTG GAC TGA CAT CGA CTT CTA TTG TT | 119 |
| siaD(C)R | GGT GTT CTC TTG TTG GGC TGT AT |  |
|  |  |  |
| siaD(Y)F | AAG GTG AAT CTT CCG AGC AGG A | 110 |
| siaD(Y)R | GAT ATC GTA CAC CAT ACC CTC TAG A |  |
|  |  |  |
| siaD(W135)L5F* | GGT GAA TCT TCC GAG CAG GA | 109 |
| siaD(W135)L5R* | GAA TAT CAT ACA CCA TGC CTT CCA TA |  |

*Primers are designed by the International Vaccine Institute laboratory

Additional. Table 2. *Neisseria Meningitidis* identifications in CSF specimens, in China, South Korea, and Vietnam, 1999 through 2002.

| **Country** | **ID** | ***ctrA*** | **Serotype**  **(Sequencing confirmed)** | **MLST** | ***crgA*** | **IS*1106*** | **16S rRNA** |
| --- | --- | --- | --- | --- | --- | --- | --- |
| Vietnam | V1 | **O** | B | **O** | NT | NT | NT |
|  | V2 | **O** | B | **O** | NT | NT | NT |
|  | V3 | **O** | C | (-) | **O** | (-) | (-) |
|  | V4 | **O** | C | (-) | **O** | (-) | (-) |
|  | V5 | **O** | C | (-) | **O** | (-) | (-) |
|  | V6 | **O** | C | (-) | **O** | (-) | (-) |
|  | V7 | **O** | C | (-) | **O** | (-) | (-) |
|  | V8 | **O** | C | (-) | (-) | **O** | (-) |
|  | V9 | **O** | C | (-) | (-) | (-) | (-) |
|  | V10 | **O** | C | (-) | **O** | (-) | (-) |
|  | V11 | **O** | C | (-) | **O** | (-) | (-) |
|  | V12 | **O** | C | (-) | **O** | (-) | (-) |
|  | V13 | **O** | C | (-) | **O** | (-) | (-) |
|  | V14 | **O** | C | (-) | **O** | (-) | (-) |
|  | V15 | **O** | C | (-) | **O** | (-) | (-) |
|  | V16 | **O** | C | (-) | **O** | (-) | (-) |
|  | V17 | **O** | C | (-) | **O** | (-) | (-) |
|  | V18 | **O** | C | (-) | **O** | (-) | (-) |
|  | V19 | **O** | C | (-) | **O** | **O** | **O** |
|  | V20 | **O** | C | (-) | (-) | **O** | **O** |
|  | V21 | **O** | C | (-) | (-) | **O** | (-) |
|  | V22 | **O** | C | (-) | (-) | **O** | (-) |
|  |  |  |  |  |  |  |  |
| China | C1 | **O** | W | (-) | (-) | (-) | (-) |
|  | C2 | **O** | Y | (-) | **O** | (-) | (-) |
|  | C3 | **O** | Y | (-) | **O** | (-) | (-) |
|  | C4 | **O** | Y | (-) | **O** | **O** | (-) |
|  | C5 | **O** | X | (-) | **O** | **O** | (-) |
|  | C6 | **O** | X | (-) | **O** | **O** | (-) |
|  | C7 | **O** | X | (-) | **O** | **O** | (-) |
|  |  |  |  |  |  |  |  |
| Korea | K1 | **O** | Y | (-) | (-) | (-) | (-) |
|  | K2 | **O** | C | (-) | (-) | **O** | (-) |
|  | K3 | **O** | Y | (-) | (-) | (-) | (-) |
|  | K4 | **O** | Y | (-) | (-) | (-) | (-) |
|  | K5 | **O** | Y | (-) | (-) | **O** | (-) |
|  | K6 | **O** | X | (-) | (-) | (-) | (-) |
|  | K7 | **O** | X | (-) | (-) | (-) | (-) |
|  | K8 | **O** | X | (-) | (-) | (-) | (-) |
|  | K9 | **O** | X | (-) | (-) | (-) | (-) |
|  | K10 | **O** | X | (-) | (-) | (-) | (-) |
|  | K11 | **O** | Y | (-) | (-) | (-) | (-) |
|  | K12 | **O** | X | (-) | (-) | (-) | (-) |
|  | K13 | **O** | X | (-) | (-) | (-) | (-) |
|  | K14 | **O** | X | (-) | (-) | (-) | (-) |
|  | K15 | **O** | X | (-) | (-) | (-) | (-) |
|  | K16 | **O** | Y | (-) | (-) | (-) | (-) |

NT: Not tested; O: PCR positive, (-): PCR negative; Multi-locus sequence typing (MLST)

Additional. Table3. Multi-locus sequence typing (MLST) results of 10 *N. meningitidis* isolates and 2 CSF specimens collected in Vietnam.

| Specimen | *abcZ* | *adk* | *aroE* | *fumC* | *gdh* | *pdhC* | *pgm* | ST |
| --- | --- | --- | --- | --- | --- | --- | --- | --- |
| Bacterial Isolate |  |  |  |  |  |  |  |  |
| VB1 | 140 | 5 | 9 | 173 | 175 | 34 | 165 | 1576 |
| VB2 | 140 | 5 | 9 | 173 | 175 | 34 | 165 | 1576 |
| VB3 | 140 | 5 | 9 | 173 | 175 | 34 | 165 | 1576 |
| VB4 | 140 | 5 | 9 | 173 | 175 | 34 | 165 | 1576 |
| VB5 | 140 | 5 | 9 | 173 | 175 | 34 | 165 | 1576 |
| VB6 | 140 | 5 | 9 | 173 | 175 | 34 | 165 | 1576 |
| VB7 | 140 | 5 | 9 | 173 | 175 | 34 | 165 | 1576 |
| VB8 | 140 | 5 | 9 | 173 | 175 | 34 | 165 | 1576 |
| VB9 | 140 | 5 | 9 | 173 | 175 | 34 | 165 | 1576 |
| VB10 | 140 | 5 | 9 | 173 | 175 | 34 | 165 | 1576 |
| CSF |  |  |  |  |  |  |  |  |
| V1 | 140 | 5 | 9 | 173 | 175 | 34 | 165 | 1576 |
| V2 | 140 | 5 | 9 | 173 | 175 | 34 | 165 | 1576 |
